# Integrated end of life care: the role of social services

**DOI:** 10.5334/ijic.1537

**Published:** 2014-03-20

**Authors:** Roberto Nuño-Solinís

**Affiliations:** O+berri, Basque Institute for Healthcare Innovation

‘To allow people the deaths they want, end of life care must be radically transformed…’. (Leadbetter C [1])

It is estimated that 75% of the population in industrialised countries will die from chronic diseases and most of them with multiple chronic conditions [2]. End of life care for multimorbid patients is particularly complex and in most health and care systems fragmented and uncoordinated. More importantly, highly medicalised and hospital-centric care often leads to overtreatment and over use of resources, but this overtreatment rarely avoids unnecessary suffering.

To address people's preferences, open conversations and advance care plans are needed. These should be flexible enough to cater for different cultural approaches to dying and to allow the implementation of appropriate care models that can improve the end of life process, increase families’ and relatives’ satisfaction and avoid unnecessary inpatient and emergency utilisation [3].

Palliative care aims to prevent and alleviate the symptoms of illness for people when curative treatment is no longer possible. This care should also address the wider psychological, social and spiritual needs of people as they approach death. Although most evidence and expertise come from palliative care for oncologic patients, this type of care is also useful for people with advanced chronic conditions and at risk of deteriorating and dying. Advances in the identification of these patients through the developments of several instruments have facilitated the implementation of palliative care in non-oncologic patients.

Although palliative care can be delivered successfully in different institutional settings such as hospitals and hospices, or at home, there is an increasing interest in the latter. Most people prefer to remain in their home at this time of their life. Various models of home-based end of life care exist, ranging from those that primarily offer nursing and personal care to others that involve multidisciplinary specialist teams. The impact of these programmes shows that more people are able to die at home as well as a reduction in the utilisation of unplanned hospital care [4]. Recent reports in the UK have covered the results of several successful and well-known models such as Marie Curie [5] and Macmillan [6].

However, these models are not widely deployed in most high-income countries and as a consequence most people with advanced diseases still die in hospitals. Although effective care integration is recognised as a key success factor for end of life care [7], many initiatives are stand-alone programmes and the potential of social support services is often neglected. In fact, palliative care has been largely considered to be a group of services provided only from health care systems and out of the social care responsibilities.

Nevertheless, social support services are perfectly positioned to help develop a more efficient and integrated model of palliative care that takes into account resources and networks (caregivers, communities, etc.) beyond the health care system.

In the Basque Country (Spain), a social innovation project provides an example of how the involvement of social support and companionship services in end of life care can achieve impressive results, such as reductions in health care utilisation estimated at 8.000 euros per case [8]. This programme called SAIATU (‘to try’ in Basque language) has filled the gap in end of life care, migrating several hospice values and skills to home care. A simple service that is fast response and family centred basing the care on the actual needs of the family and empowering them in caring for their loved one facilitates adaptation to the different stages of the illness through a social, emotional approach. The key is 24/7 communication, with a one face to one family motto. This is an integrated care model that reaches out and acts as a bridge working alongside the existing resources and filling in the gaps between standard health care and social services.

If palliative care must be holistic, then it should include the social nature of the aid. Many of the effective solutions that are often required to adequately take care of each case are not health services but rather social ones. If these social resources and benefits are not offered, it will mean greater costs for the health system and a dysfunctional use of the competences of health care professionals for tackling social needs that are better addressed by other types of professionals. The integration of social support in the provision of palliative care seems to be an efficient way to respond to the complex mix of needs of people in the end stage of their lives, allowing them to die according to their preferences and at the same time contributing to the sustainability of welfare systems.

## References

[1] Leadbeater C, Garber J (2010). Dying for change.

[2] Gómez-Batiste X, Martínez-Muñoz M, Blay C, Espinosa J, Contel JC, Ledesma A (2012). Identifying needs and improving palliative care of chronically ill patients: a community-oriented, population-based, public-health approach. Current Opinion in Supportive and Palliative Care.

[3] Brumley R, Enguidanos S, Jamison P, Seitz R, Morgenstern N, Saito S (2007). Increased satisfaction with care and lower costs: results of a randomized trial of in-home palliative care. Journal of the American Geriatrics Society.

[4] Gomes B, Calanzani N, Curiale V, McCrone P, Higginson IJ (2013). Effectiveness and cost-effectiveness of home palliative care services for adults with advanced illness and their caregivers. The Cochrane Database of Systematic Reviews.

[5] Chitnis X, Georghiou T, Steventon A, Bardsley M (2012). The impact of the Marie Curie Nursing service on place of death and hospital use at the end of life.

[6] Thiel V, Sonola L, Goodwin N, Kodner DL (2013). Midhurst Macmillan Community Specialist Palliative Care Service. Delivering end of live care in the community.

[7] Integrated End-of-Life Care ∣AHRQ Innovations Exchange [webpage on the internet].

[8] The SAIATU project (2012). Impact of the provision of social care within a Palliative Care Programme on healthcare costs.

